# Supplementary material for: Improved cohesin HiChIP protocol and bioinformatic analysis for robust detection of chromatin loops and stripes
Source: Commun Biol. 2025 Mar 14;8:437. doi: 10.1038/s42003-025-07847-w (PMC11906747; doi:10.1038/s42003-025-07847-w)
Supplement: Supplementary file 2 — Supplementary Information [file 42003_2025_7847_MOESM2_ESM.pdf]

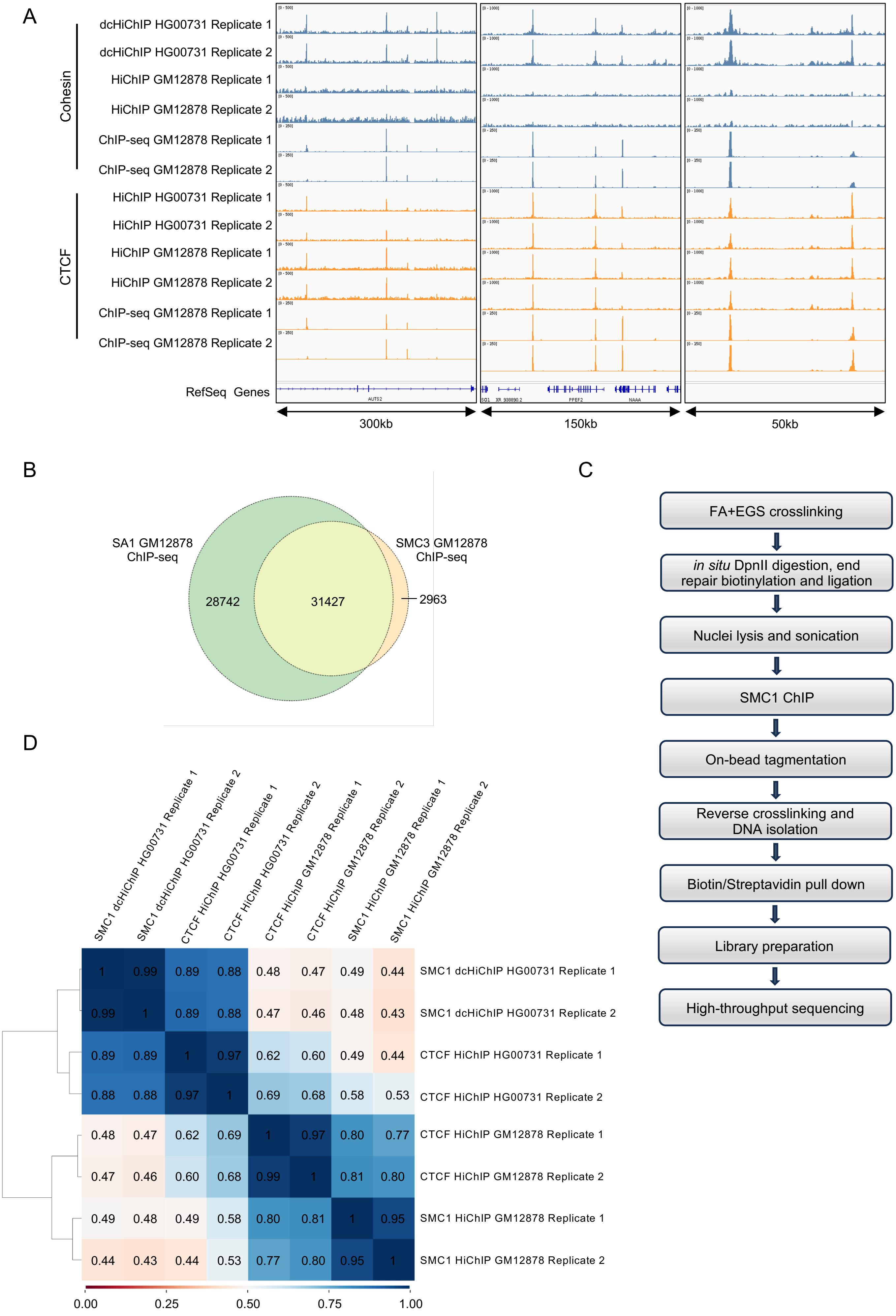
A
Cohesin
dcHiChIP HG00731 Replicate 1
dcHiChIP HG00731 Replicate 2
HiChIP GM12878 Replicate 1
HiChIP GM12878 Replicate 2
ChIP-seq GM12878 Replicate 1
ChIP-seq GM12878 Replicate 2
CTCF
HiChIP HG00731 Replicate 1
HiChIP HG00731 Replicate 2
HiChIP GM12878 Replicate 1
HiChIP GM12878 Replicate 2
ChIP-seq GM12878 Replicate 1
ChIP-seq GM12878 Replicate 2
RefSeq Genes
AUTS2
PPEF2
NAAA
300kb
150kb
50kb
B
SA1 GM12878 ChIP-seq
SMC3 GM12878 ChIP-seq
28742
31427
2963
C
FA+EGS crosslinking
in situ DpnII digestion, end repair biotinylation and ligation
Nuclei lysis and sonication
SMC1 ChIP
On-bead tagmentation
Reverse crosslinking and DNA isolation
Biotin/Streptavidin pull down
Library preparation
High-throughput sequencing
D
SMC1 dcHiChIP HG00731 Replicate 1
SMC1 dcHiChIP HG00731 Replicate 2
CTCF HiChIP HG00731 Replicate 1
CTCF HiChIP HG00731 Replicate 2
CTCF HiChIP GM12878 Replicate 1
CTCF HiChIP GM12878 Replicate 2
SMC1 HiChIP GM12878 Replicate 1
SMC1 HiChIP GM12878 Replicate 2
1 0.99 0.89 0.88 0.48 0.47 0.49 0.44 SMC1 dcHiChIP HG00731 Replicate 1
0.99 1 0.89 0.88 0.47 0.46 0.48 0.43 SMC1 dcHiChIP HG00731 Replicate 2
0.89 0.89 1 0.97 0.62 0.60 0.49 0.44 CTCF HiChIP HG00731 Replicate 1
0.88 0.88 0.97 1 0.69 0.68 0.58 0.53 CTCF HiChIP HG00731 Replicate 2
0.48 0.47 0.62 0.69 1 0.97 0.80 0.77 CTCF HiChIP GM12878 Replicate 1
0.47 0.46 0.60 0.68 0.99 1 0.81 0.80 CTCF HiChIP GM12878 Replicate 2
0.49 0.48 0.49 0.58 0.80 0.81 1 0.95 SMC1 HiChIP GM12878 Replicate 1
0.44 0.43 0.44 0.53 0.77 0.80 0.95 1 SMC1 HiChIP GM12878 Replicate 2
0.00
0.25
0.50
0.75
1.00

**Supplementary Figure 1**. **A.** IGV browser coverage tracks from separate replicates of the HiChIP and ChIP-seq experiments analysed in this study. The following example genomic regions are shown: (1) 300 kb window - chr7:69,997,000-70,297,300; (2) 150 kb window - chr4:75,809,763-75,960,323; (3) 50 kb window - chr7:4,600,000-4,650,000. **B.** Venn diagrams showing common peaks between GM12878 SA1 ChIP-seq and GM12878 SMC3 ChIP-seq. **C.** Schematic representation of the experimental steps performed during the cohesin FA-EGS HiChIP protocol. D. Heatmap showing Pearson correlation coefficient between the individual replicates of the HiChIP and ChIP-seq samples processed in this study.

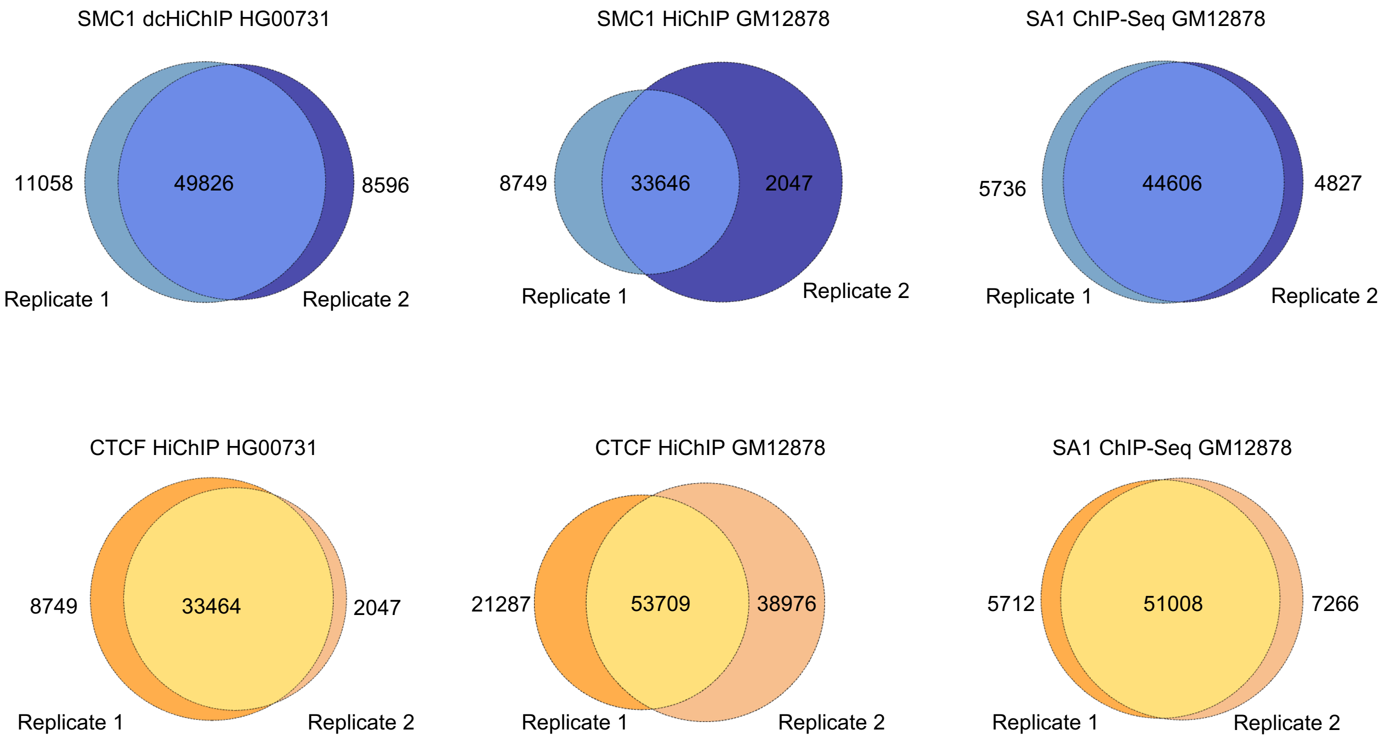


**Supplementary Figure 2.** Venn diagrams showing common peaks between peaks called in the first and second replicate for the indicated HiChIP and ChIP-seq experiments.

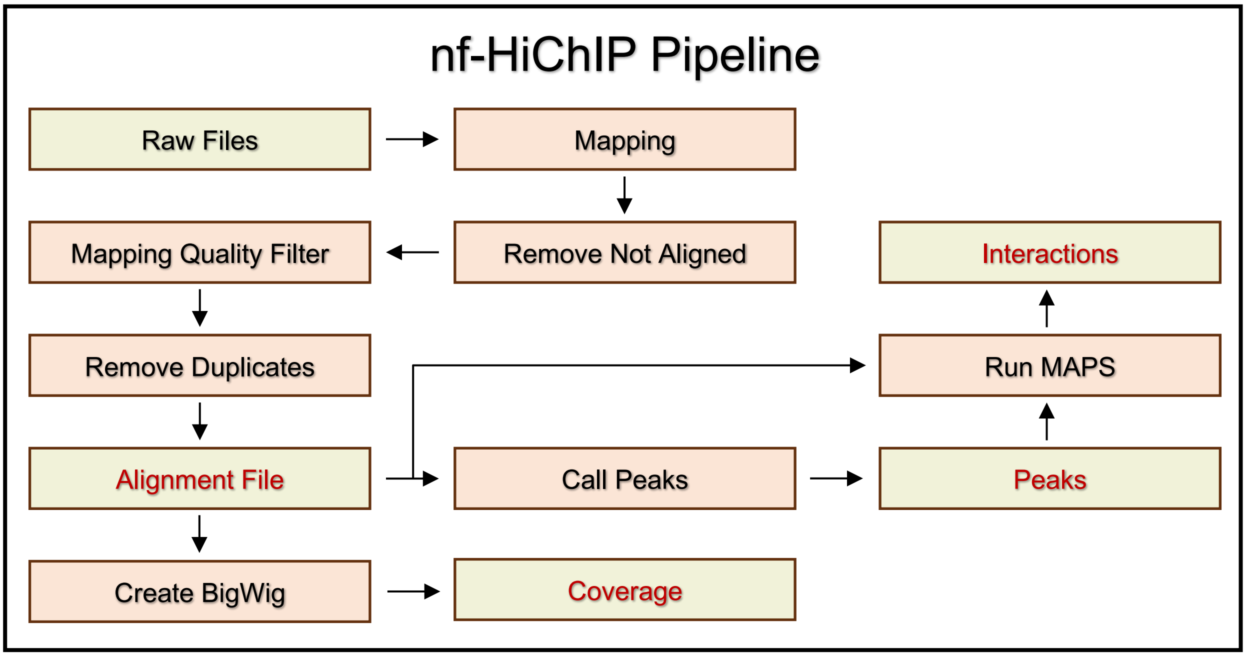


**Supplementary Figure 3.** nf-HiChIP workflow.

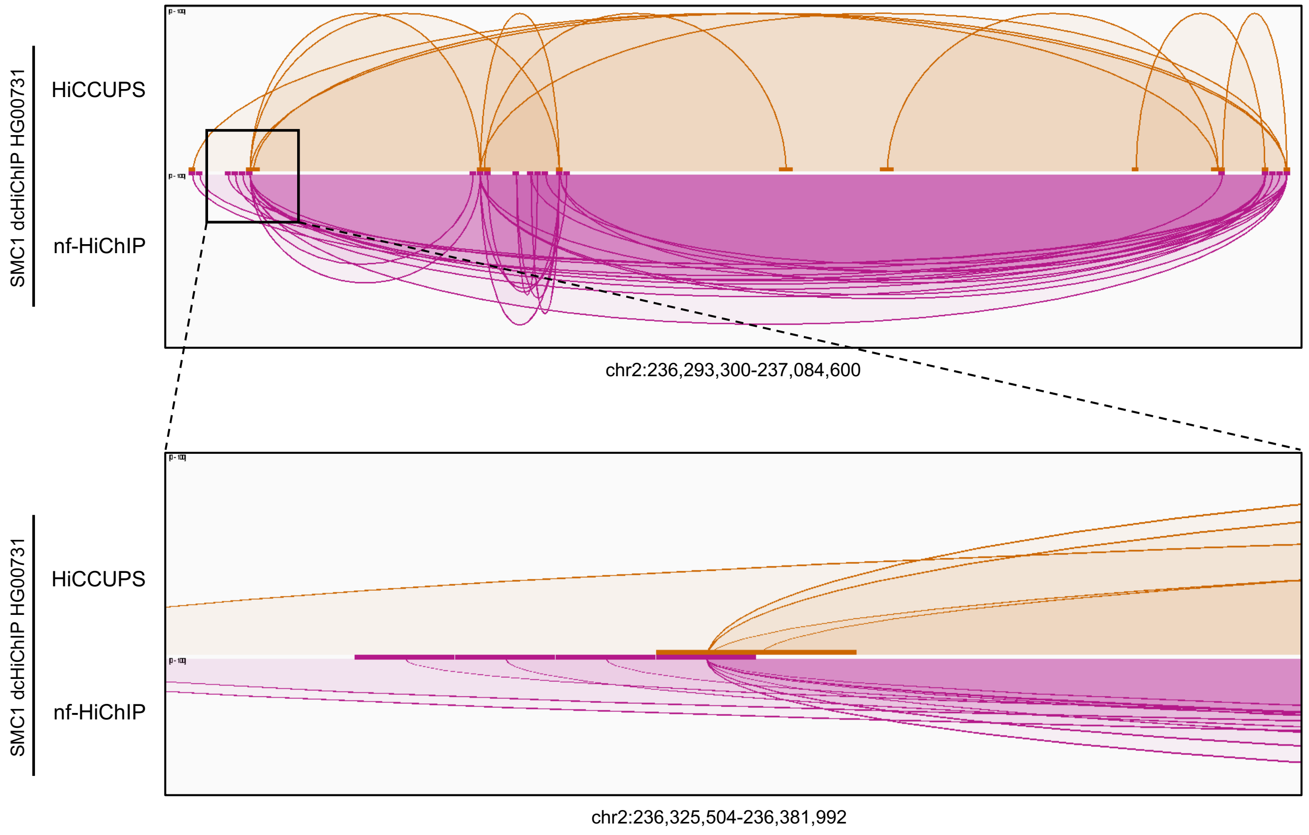


**Supplementary Figure 4.** HiCCUPS uses an image processing approach to identify loops (shown as yellow) from chromatin interaction maps at three different resolutions: 5 kb, 10 kb, and 25 kb and then merges loops based on the predefined threshold. In contrast, as nf-HiChIP directly detects loops from interaction data at a single, user-defined resolution 5 kb (as shown in red). Due to these methodological differences specifically the higher resolution and lack of merging in nf-HiChIP - multiple nf-HiChIP loops anchors can overlap with a single HiCCUPS loop anchors.

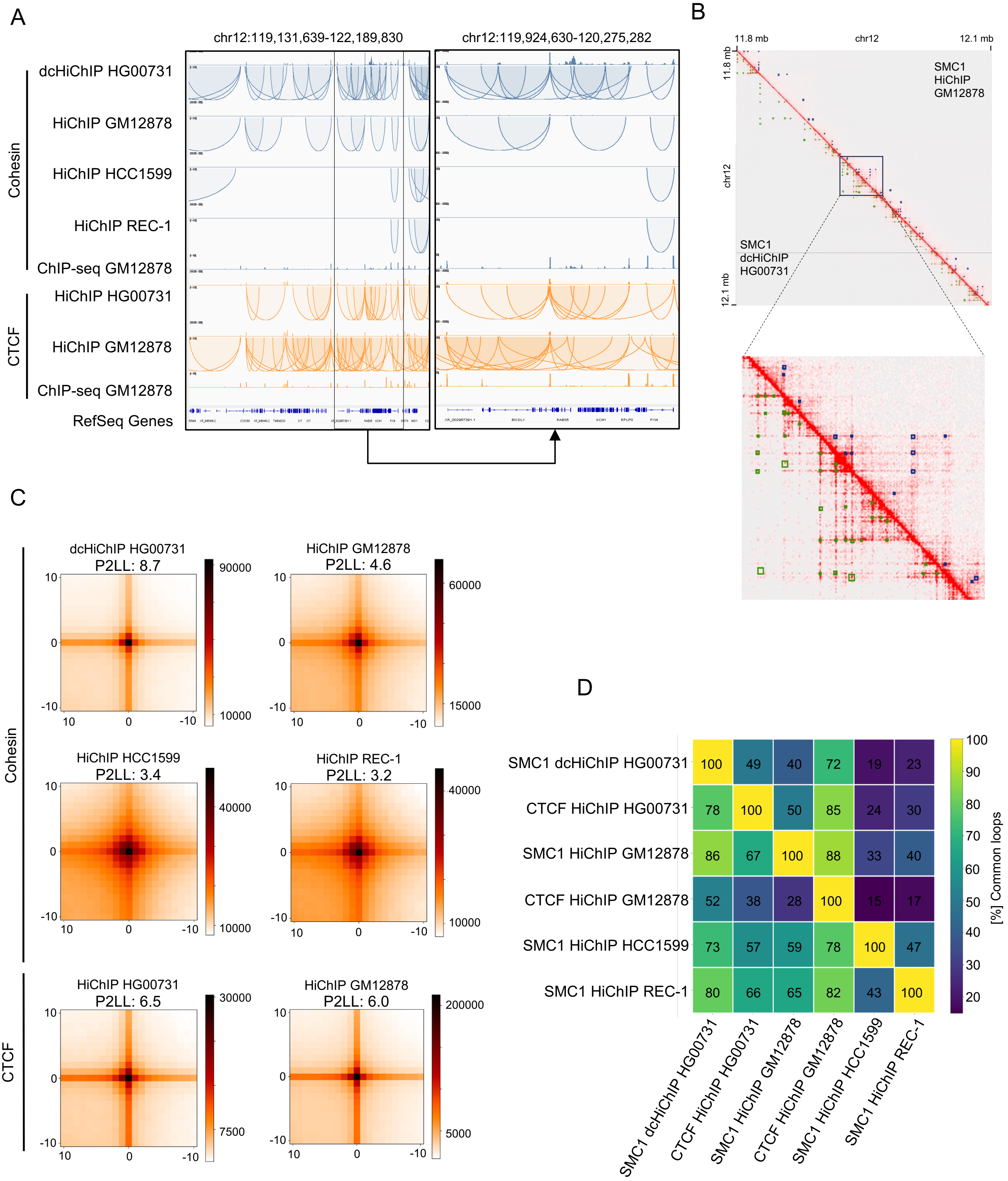
A
chr12:119,131,639-122,189,830
chr12:119,924,630-120,275,282
Cohesin
dcHiChIP HG00731
HiChIP GM12878
HiChIP HCC1599
HiChIP REC-1
ChIP-seq GM12878
CTCF
HiChIP HG00731
HiChIP GM12878
ChIP-seq GM12878
RefSeq Genes
B
11.8 mb
chr12
12.1 mb
SMC1
HiChIP
GM12878
SMC1
dcHiChIP
HG00731
C
Cohesin
dcHiChIP HG00731
P2LL: 8.7
90000
10000
HiChIP GM12878
P2LL: 4.6
60000
15000
HiChIP HCC1599
P2LL: 3.4
40000
10000
HiChIP REC-1
P2LL: 3.2
40000
10000
CTCF
HiChIP HG00731
P2LL: 6.5
30000
7500
HiChIP GM12878
P2LL: 6.0
200000
5000
10
0
-10
D
SMC1 dcHiChIP HG00731: 100 49 40 72 19 23
CTCF HiChIP HG00731: 78 100 50 85 24 30
SMC1 HiChIP GM12878: 86 67 100 88 33 40
CTCF HiChIP GM12878: 52 38 28 100 15 17
SMC1 HiChIP HCC1599: 73 57 59 78 100 47
SMC1 HiChIP REC-1: 80 66 65 82 43 100
SMC1 dcHiChIP HG00731
CTCF HiChIP HG00731
SMC1 HiChIP GM12878
CTCF HiChIP GM12878
SMC1 HiChIP HCC1599
SMC1 HiChIP REC-1
[%] Common loops
100
90
80
70
60
50
40
30
20

**Supplementary Figure 5. FA-EGS cross-linking HiChIP protocol improves detection of cohesin-mediated loops (HICCUPS). A.** IGV browser coverage tracks and HICCUPS loops of indicated HiChIP samples and coverage tracks for SA1 and CTCF GM12878 ChIP-seq experiments. **B.** Juicebox interaction maps at 5 kb resolution of the example region for the indicated cohesin HiChIP samples. Loops (HICCUPS) are shown as green (SMC1 dcHiChIP HG00731) or blue (SMC1 HiChIP GM12878) rectangles. **C.** Aggregate Peak Analysis (APA) performed using the loops identified by HICCUPS for the indicated cohesin and CTCF HiChIP samples. APA score P2LL (peak to left lower corner) is the ratio of the central bin to the average of the lower left corner and indicates the strength of the loop. **D.** Heatmap showing the HiCCUPS loop overlap with 15 kb tolerance between cohesin and CTCF HiChIP samples**.**

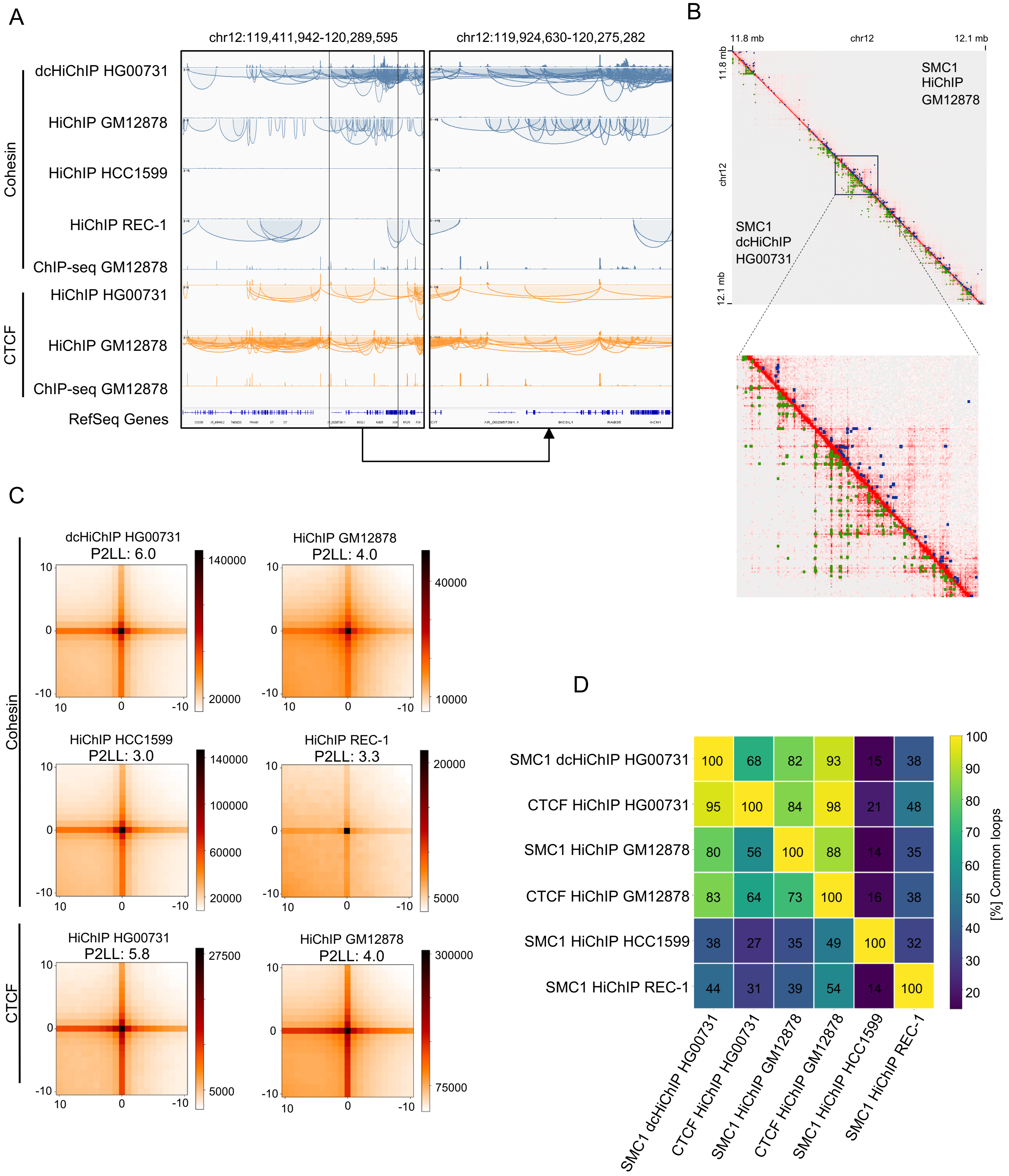

A
chr12:119,411,942-120,289,595
chr12:119,924,630-120,275,282
Cohesin
dcHiChIP HG00731
HiChIP GM12878
HiChIP HCC1599
HiChIP REC-1
ChIP-seq GM12878
CTCF
HiChIP HG00731
HiChIP GM12878
ChIP-seq GM12878
RefSeq Genes
B
11.8 mb
chr12
12.1 mb
SMC1
HiChIP
GM12878
SMC1
dcHiChIP
HG00731
C
Cohesin
dcHiChIP HG00731
P2LL: 6.0
HiChIP GM12878
P2LL: 4.0
HiChIP HCC1599
P2LL: 3.0
HiChIP REC-1
P2LL: 3.3
CTCF
HiChIP HG00731
P2LL: 5.8
HiChIP GM12878
P2LL: 4.0
D
SMC1 dcHiChIP HG00731 100 68 82 93 15 38
CTCF HiChIP HG00731 95 100 84 98 21 48
SMC1 HiChIP GM12878 80 56 100 88 14 35
CTCF HiChIP GM12878 83 64 73 100 16 38
SMC1 HiChIP HCC1599 38 27 35 49 100 32
SMC1 HiChIP REC-1 44 31 39 54 14 100
SMC1 dcHiChIP HG00731
CTCF HiChIP HG00731
SMC1 HiChIP GM12878
CTCF HiChIP GM12878
SMC1 HiChIP HCC1599
SMC1 HiChIP REC-1
[%] Common loops

**Supplementary Figure 6. FA-EGS cross-linking HiChIP protocol improves detection of cohesin-mediated loop detection (ChIA-PIPE). A.** IGV genome browser view showing coverage tracks and ChIA-PIPE loops of the indicated HiChIP samples and coverage tracks for SA1 and CTCF GM12878 ChIP-seq experiments. **B.** Juicebox interaction maps at 5 kb resolution of the example region for the indicated cohesin HiChIP samples. Loops (ChIA-PIPE) are shown as green (SMC1 HiChIP HG00731) or blue (SMC1 HiChIP GM12878) rectangles. **C.** Aggregate Peak Analysis (APA) performed using the loops identified by ChIA-PIPE for the indicated cohesin and CTCF HiChIP samples. APA score P2LL (peak to left lower corner) is the ratio of the central bin to the average of the lower left corner and indicates the strength of the loop. **D.** Heatmap showing the ChIA-PIPE loop overlap with 15 kb tolerance between cohesin and CTCF HiChIP samples.

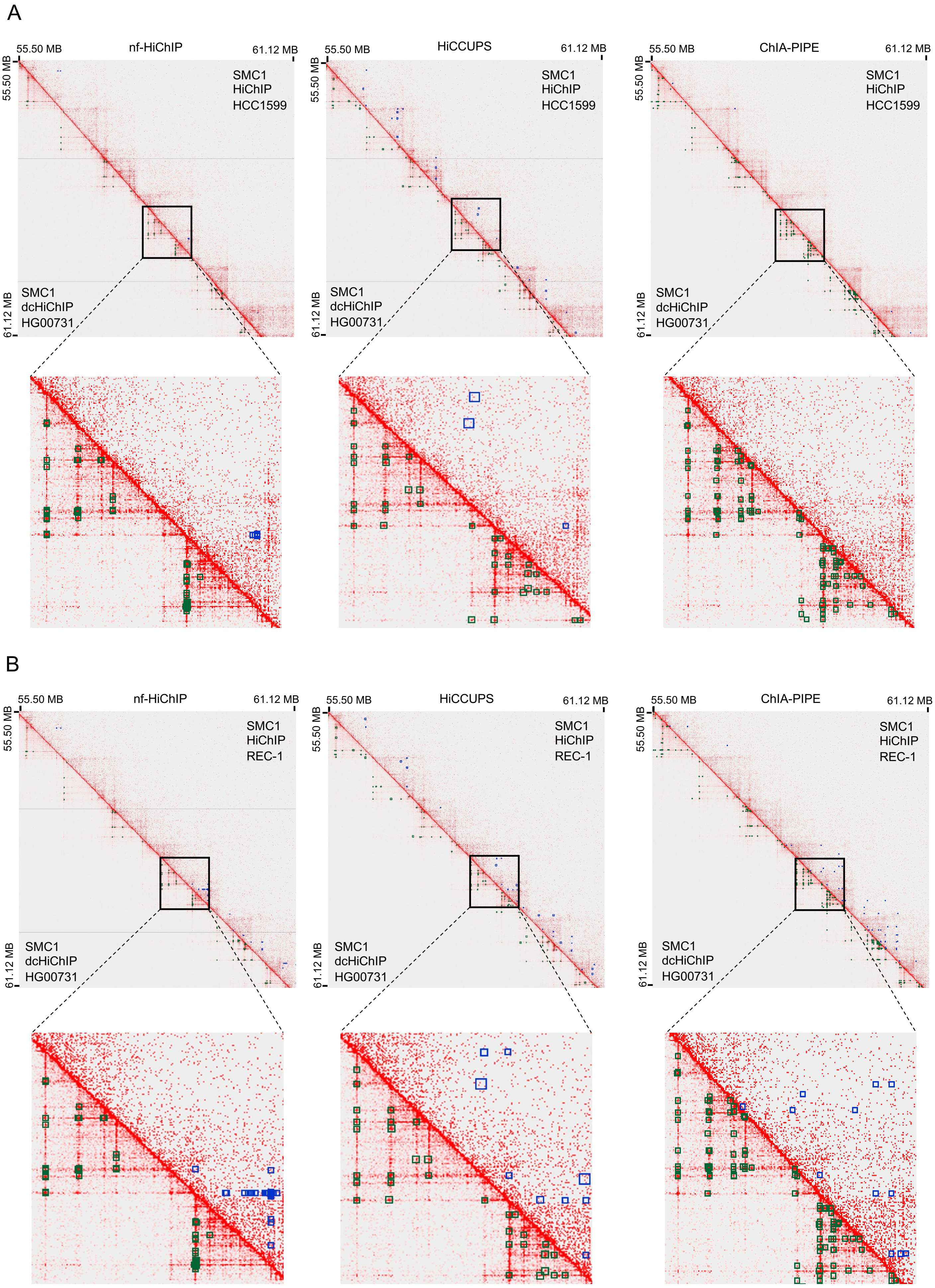

A
55.50 MB
nf-HiChIP
61.12 MB
55.50 MB
SMC1
HiChIP
HCC1599
SMC1
dcHiChIP
HG00731
61.12 MB
HiCCUPS
ChIA-PIPE
B
SMC1
HiChIP
REC-1

**Supplementary Figure 7. A.** Juicebox interaction maps at 5 kb resolution of the example region (chr14:55,500,000-61,120,000) for the indicated cohesin HiChIP samples. Loops (nf-HiChIP, HiCCUPS and ChIA-PIPE) are shown as green (SMC1 HiChIP HG00731) or blue (SMC1 HiChIP HCC1599) rectangles. **B.** Juicebox interaction maps at 5 kb resolution of the example region (chr14:55,500,0001-61,120,000) for the indicated cohesin HiChIP samples. Loops (nf-HiChIP, HiCCUPS and ChIA-PIPE) are shown as green (SMC1 HiChIP HG00731) or blue (SMC1 HiChIP REC-1) rectangles.

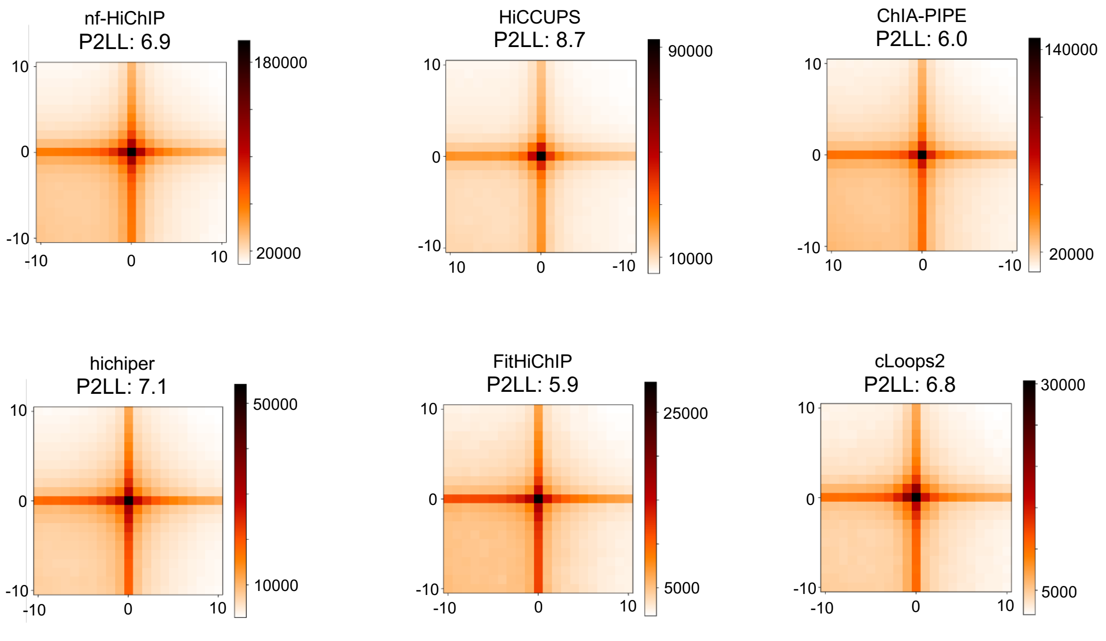


**Supplementary Figure 8.** APA analysis performed using loops identified different loop calling algorithms – nf-HiChIP, HiCCUPS, ChIA-PIPE, hichiper, FitHiChIP, cLoops2 on SMC1 HiChIP HG00731 sample.

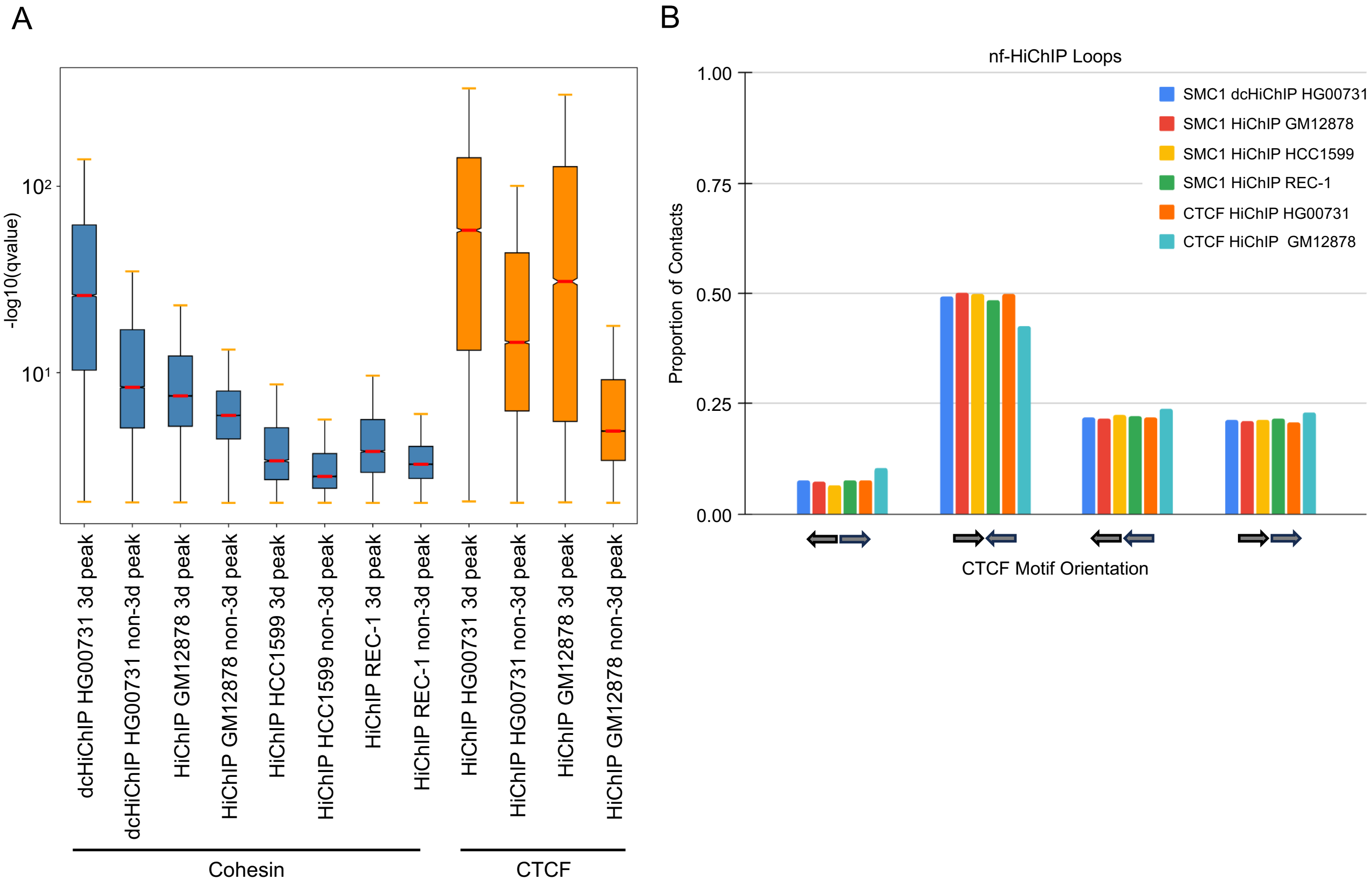


**Supplementary Figure 9. A.** Distribution of -log(10) qvalue of peaks called by MACS3 in the indicated samples. Peaks that are localised at the loop anchors are marked by 3d peaks and those not localised at the anchors are marked by non-3d peak. The red horizontal line inside each boxplot indicates the median. The bottom edge of the box marks the first quartile, and the top edge marks the third quartile and the whiskers extend to the minimum and maximum values, excluding outliers, which are not shown. **B**. CTCF motif orientation analysis at HiChIP contact anchors in all the samples. Here, "←→" is opposite, "→←" is convergent, "←←" is tandem left, and "→→" is tandem left motif orientation.

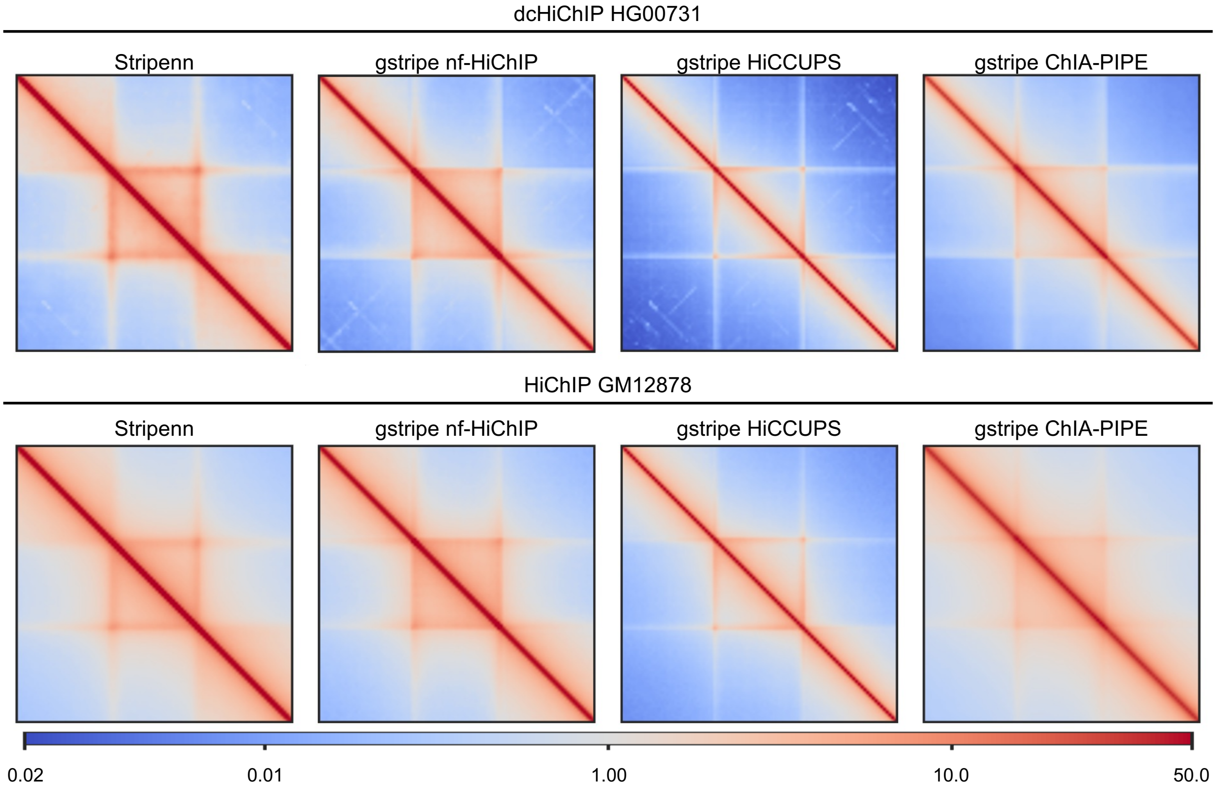


**Supplementary Figure 10.** Pileup plots constructed for stripe regions display averaged contact map values within the stripe domains for cohesin HiChIP samples. Upper and lower panel present SMC1 FA-EGS HiChIP (HG00731) and SMC1 HiChIP (GM12878), respectively and stripes were called by the algorithms indicated above the pileup plots. Coolpuppy API was used to obtain the plots, with parameters equivalent to using "--rescale --local --unbalanced" options, i.e. each stripe domain was expanded by regions of equal size from both sides, then rescaled to a standard size, and the the raw contact matrix signal is averaged over all stripe domains. The color scale is logarithmic.

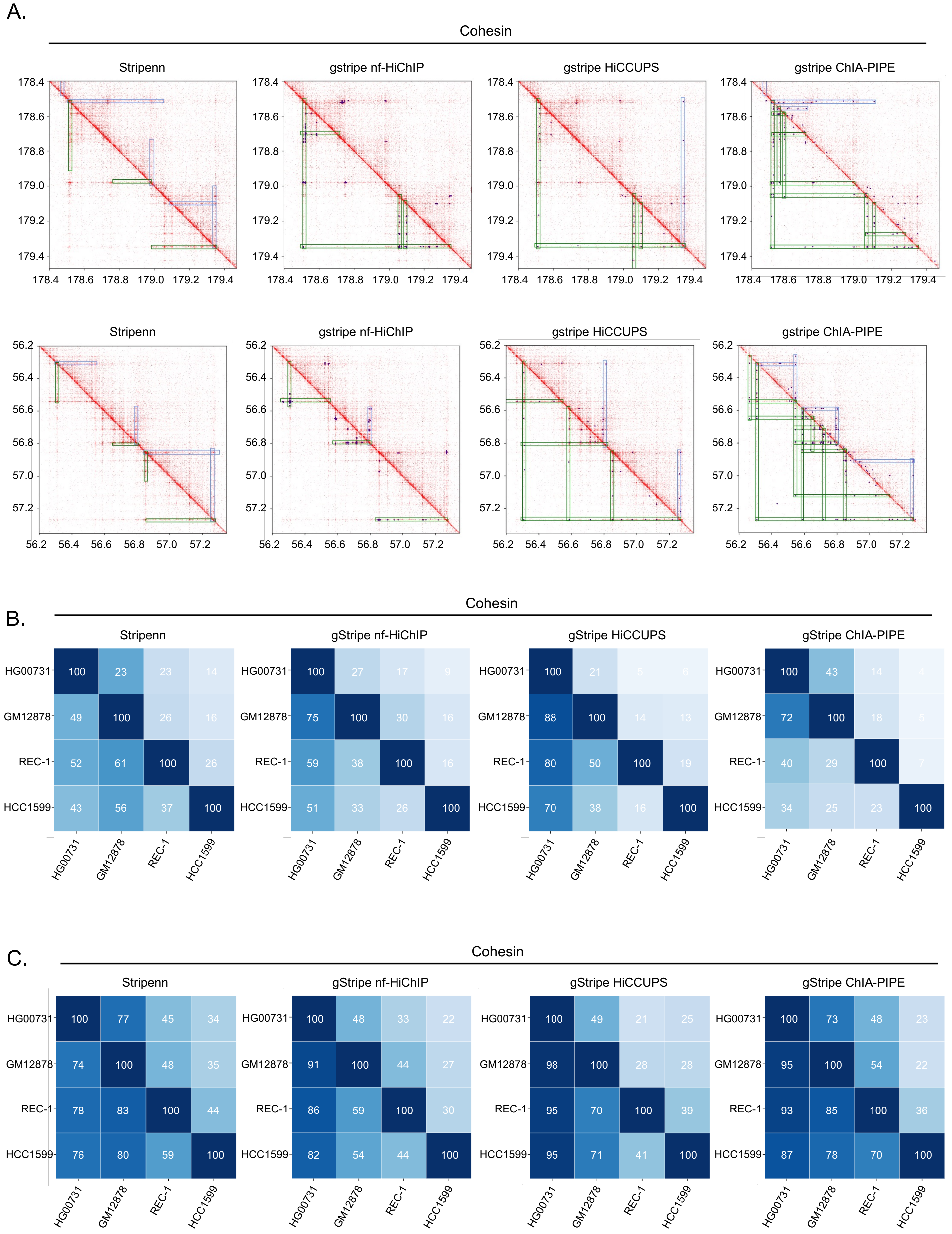
A.
Cohesin
Stripenn
gstripe nf-HiChIP
gstripe HiCCUPS
gstripe ChIA-PIPE
178.4 178.6 178.8 179.0 179.2 179.4
56.2 56.4 56.6 56.8 57.0 57.2
B.
Cohesin
Stripenn
gStripe nf-HiChIP
gStripe HiCCUPS
gStripe ChIA-PIPE
HG00731 GM12878 REC-1 HCC1599
HG00731 100 23 23 14 | 100 27 17 9 | 100 21 5 6 | 100 43 14 4
GM12878 49 100 26 16 | 75 100 30 16 | 88 100 14 13 | 72 100 18 5
REC-1 52 61 100 26 | 59 38 100 16 | 80 50 100 19 | 40 29 100 7
HCC1599 43 56 37 100 | 51 33 26 100 | 70 38 16 100 | 34 25 23 100
C.
Cohesin
Stripenn
gStripe nf-HiChIP
gStripe HiCCUPS
gStripe ChIA-PIPE
HG00731 GM12878 REC-1 HCC1599
HG00731 100 77 45 34 | 100 48 33 22 | 100 49 21 25 | 100 73 48 23
GM12878 74 100 48 35 | 91 100 44 27 | 98 100 28 28 | 95 100 54 22
REC-1 78 83 100 44 | 86 59 100 30 | 95 70 100 39 | 93 85 100 36
HCC1599 76 80 59 100 | 82 54 44 100 | 95 71 41 100 | 87 78 70 100

**Supplementary Figure 11. A.** Comparison of stripes called on our HiChIP data (HG00731, below diagonal, green) and the GM12878 data (above diagonal, blue) for two additional example regions: chr1:178,421,512-179,491,193 (top) and chr6:56,250,000-57,400,000 (bottom). The first three columns show stripes called using gStripe with loops (shown as purple dots) obtained using nf-HiChIP, ChIA-PIPE and HiCCUPS, respectively. The last column shows stripes called by Stripenn. **B**. Overlap between stripe anchors called in each dataset by gStripe (using loops obtained from nf-HiChIP, HiCCUPS or ChIA-PIPE) and Stripenn. A single cell in the matrix represents the percentage of stripes in the row sample overlapping one or more stripes in the column sample. For example, in the first panel, 72% of the stripes from the GM12878 (row 2) are present in HG00731 (column 1). **C**. Overlap between stripe domains. A stripe domain refers to the entire length of the stripe, not just the anchor. Overlapping domains are merged. The datasets and matrix construction are the same as in B.

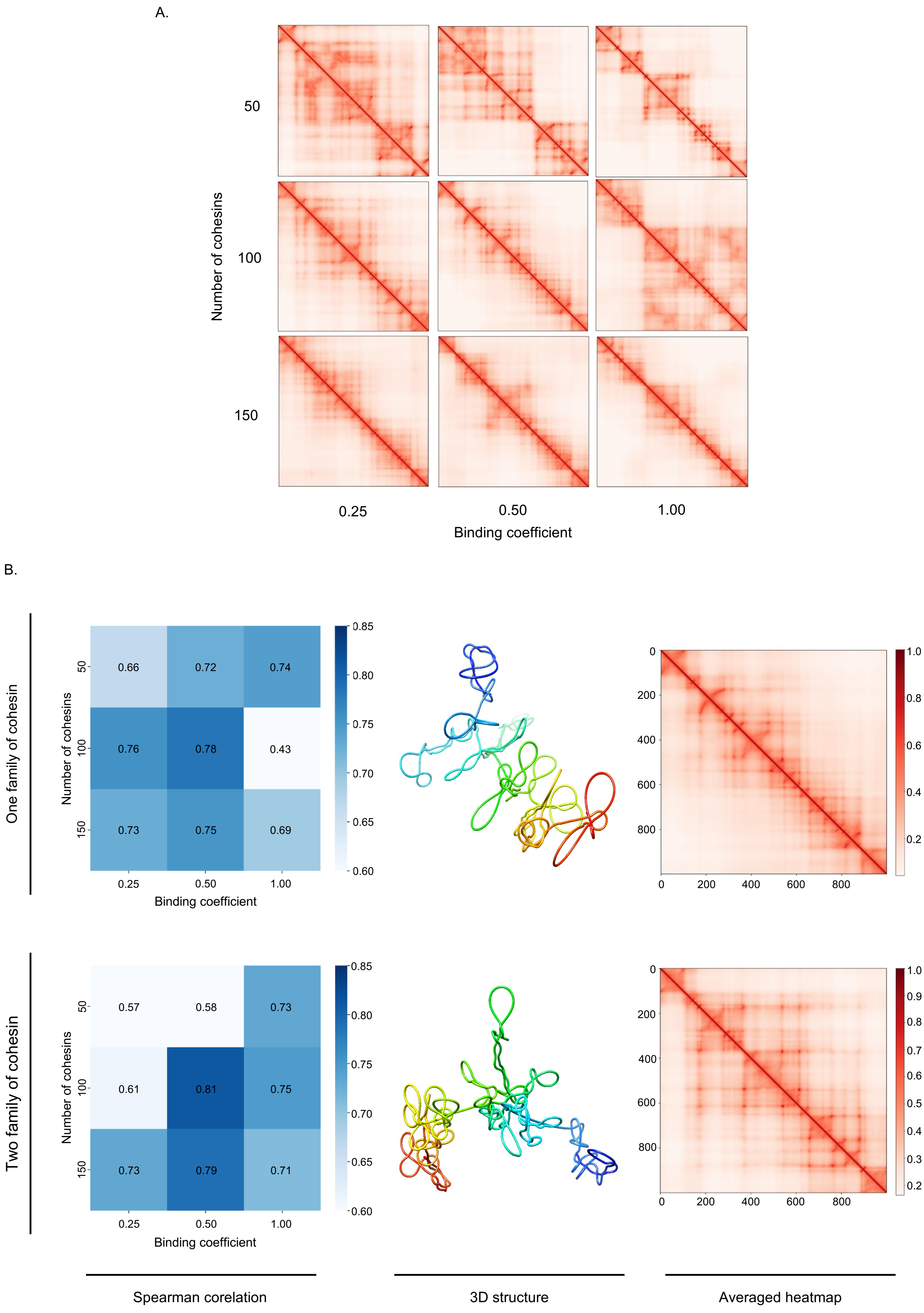
A.
Number of cohesins
50
100
150
0.25
0.50
1.00
Binding coefficient
B.
One family of cohesin
Number of cohesins
50
100
150
0.66
0.72
0.74
0.76
0.78
0.43
0.73
0.75
0.69
0.25
0.50
1.00
Binding coefficient
0.85
0.80
0.75
0.70
0.65
0.60
0
200
400
600
800
1.0
0.8
0.6
0.4
0.2
Two family of cohesin
Number of cohesins
50
100
150
0.57
0.58
0.73
0.61
0.81
0.75
0.73
0.79
0.71
0.25
0.50
1.00
Binding coefficient
0.85
0.80
0.75
0.70
0.65
0.60
0
200
400
600
800
1.0
0.9
0.8
0.7
0.6
0.5
0.4
0.3
0.2
Spearman corelation
3D structure
Averaged heatmap

**Supplementary Figure 12: Choosing the appropriate biophysical model for the exemplary region (chr1:178421513-179491193)**. **A.** The resulting average inverse distance heatmaps from the ensemble of 3D structures for different values of the number of cohesins and the binding coefficient. We see that we can either have well confined borders with only short-range loops and stripes with high values of binding coefficient and number of cohesins, or long-range patterns without clear borders with small amount of cohesins and small binding coefficients. **B.** (Left) The first column shows Spearman correlation between simulated and experimental heatmap with only one family of cohesins, and two families of extruders. (Middle) The second column shows the final 3D structures. (Right) The third column present averaged inverse distance heatmaps from the ensemble of structures. In both simulations, the same biophysical parameters were used (100 cohesins, binding coefficient = 0.5). For the second cohesin family, five cohesins were assigned a folding coefficient 10 times higher than that of the first family.

<table>
<tr><th>Human cell line</th><th>Cell Type</th><th>Target protein</th><th>Cross-linking agent</th><th>Source</th><th>Acession Id</th></tr>
<tr><td>dcHiChIP HG00731</td><td>LCL*</td><td rowspan="4">Cohesin (SMC1)</td><td>FA + EGS</td><td>This study</td><td>GSE266640</td></tr>
<tr><td>HiChIP GM12878</td><td>LCL*</td><td rowspan="6">FA</td><td>Mumbach et al., 2016</td><td>GSE80820</td></tr>
<tr><td>HiChIP HCC1599</td><td>Epithelial</td><td rowspan="2">Petrovic et al, 2019</td><td>GSE116872</td></tr>
<tr><td>HiChIP REC-1</td><td>Lymphoblast</td><td>GSE116875</td></tr>
<tr><td>ChIP-seq GM12878</td><td>LCL*</td><td>Cohesin (SA1)</td><td rowspan="2">Kasowski, Maya et al., 2013</td><td>GSE50893</td></tr>
<tr><td>ChIP-seq GM12878</td><td>LCL*</td><td rowspan="3">CTCF</td><td>GSE50893</td></tr>
<tr><td>HiChIP HG00731</td><td>LCL*</td><td>This study</td><td>GSE266640</td></tr>
<tr><td>HiChIP GM12878</td><td>LCL*</td><td>Glutaraldehyde</td><td>Mumbach et al., 2016</td><td>GSE115524</td></tr>
</table>

*LCL from 1000 Genome Project (1000 Genomes Project Consortium et al., 2015)

**Supplementary Table 1.** HiChIP experiments included in the study.

| Dataset | Type of experiment | Number of replicates | Number of reads | Number of peaks | 3D peaks | Non 3D peaks | % of 3D peaks |
|---|---|---|---|---|---|---|---|
| SMC1 HG00731 | dcHiCHIP | 2 | 607,768,604 | 97364 | 49638 | 47726 | 50.98188242 |
| HG00731 CTCF | HiCHIP | 2 | 303,575,305 | 63555 | 17625 | 45930 | 27.73188577 |
| SMC1 GM12878 | HiCHIP | 2 | 643,644,994 | 666380 | 74887 | 591493 | 11.23788229 |
| CTCF GM12878 | HiCHIP | 2 | 533,531,475 | 153256 | 23405 | 129851 | 15.27183275 |
| SMC1 HCC1599 | HiCHIP | 1 | 769,438,941 | 21281 | 8371 | 26008 | 39.33555754 |
| SMC1 REC-1 | HiCHIP | 1 | 762,451,158 | 34379 | 5968 | 15313 | 17.35943454 |
| SA1 GM12878 | ChIP-seq | 2 | 57,401,728 | 60169 | - | - | - |
| CTCF GM12878 | ChIP-seq | 2 | 52,588,683 | 67179 | - | - | - |

**Supplementary Table 2.** Number of biological replicates, total number of reads and MACS3 peaks for indicated experiments.

| | CTCF ChIP-seq peaks | CTCF HiChIP peaks |
|---|---|---|
| **CTCF ChIP-seq peaks** | 100% | 95% |
| **CTCF HiChIP peaks** | 98% | 100% |

**Supplementary Table 3.** Overlap between CTCF HiChIP loops sets detected with MAPS using CTCF ChIP-seq peaks or CTCF HiChIP peaks.

| Dataset | HiCCUPS in nf-HiChIP | | | | nf-HiChIP in HiCCUPS | | | |
|---|---|---|---|---|---|---|---|---|
| | Strict overlap | | 15kb tolarance | | Strict overlap | | 15kb tolerance | |
| SMC1 dcHiChIP HG00731 | 19387 | 44.93% | 20243 | 46.92% | 45029 | 55.95% | 71349 | 88.65% |
| CTCF HiChIP HG00731 | 6912 | 25.99% | 7423 | 27.92% | 14559 | 51.97% | 24173 | 86.30% |
| SMC1 HiChIP GM12878 | 7417 | 38.08% | 7688 | 39.47% | 19000 | 52.00% | 32085 | 87.82% |
| CTCF HiChIP GM12878 | 24398 | 40.83% | 26236 | 43.91% | 65081 | 36.45% | 138024 | 77.31% |
| SMC1 HiChIP HCC1599 | 2890 | 26.81% | 3065 | 28.43% | 7084 | 41.37% | 13298 | 77.66% |
| SMC1 HiChIP REC-1 | 3843 | 32.65% | 4163 | 35.36% | 8897 | 39.91% | 16666 | 74.76% |

**Supplementary Table 4.** Overlap between loops (number and percentage) between the HiCCUPS and nf-HiChIP.

| Dataset | nf-HiChIP | HiCCUPS | ChIA-PIPE | hichiper | FitHiChIP | cLoops2 |
|---|---|---|---|---|---|---|
| SMC1 dcHiChIP HG00731 | 80487 | 43148 | 179516 | 80487 | 127278 | 164377 |
| CTCF HiChIP HG00731 | 28012 | 26590 | 46117 | -- | -- | -- |
| SMC1 HiChIP GM12878 | 36535 | 19478 | 92248 | -- | -- | -- |
| CTCF HiChIP GM12878 | 178535 | 59753 | 236997 | -- | -- | -- |
| SMC1 HiChIP HCC1599 | 17124 | 10779 | 19057 | -- | -- | -- |
| SMC1 HiChIP REC-1 | 22294 | 11772 | 46112 | -- | -- | -- |

**Supplementary Table 5.** Number of significant inetractions (loops) detected by three independent algorithms: nf-HiChIP, HICCUPS and ChIA-PIPE.

| Loops Calling Pipeline | Dataset | ←→ | →← | ←← | →→ | ←. | →. | .← | .→ | None |
|---|---|---|---|---|---|---|---|---|---|---|
| nf-HiChIP | SMC1 dcHiChIP HG00731 | 5173 | 33271 | 14682 | 14431 | 1520 | 4864 | 4438 | 1571 | 537 |
| | CTCF HiChIP HG00731 | 1881 | 12397 | 5447 | 5189 | 309 | 1149 | 1300 | 311 | 29 |
| | SMC1 HiChIP GM12878 | 2208 | 15421 | 6673 | 6435 | 711 | 2087 | 2122 | 639 | 239 |
| | CTCF HiChIP GM12878 | 15243 | 61912 | 34776 | 33252 | 4270 | 11769 | 12265 | 4040 | 1008 |
| | SMC1 HiChIP HCC1599 | 947 | 7290 | 3259 | 3093 | 212 | 966 | 1032 | 247 | 78 |
| | SMC1 HiChIP REC-1 | 1445 | 9090 | 4177 | 4073 | 408 | 1288 | 1334 | 360 | 119 |
| | | | | | | | | | | |
| HiCCUPS | SMC1 dcHiChIP HG00731 | 2910 | 20850 | 7662 | 7785 | 533 | 1186 | 1378 | 607 | 228 |
| | CTCF HiChIP HG00731 | 2405 | 12344 | 5224 | 5105 | 237 | 479 | 533 | 243 | 11 |
| | SMC1 HiChIP GM12878 | 1175 | 9997 | 3347 | 3460 | 186 | 474 | 511 | 240 | 79 |
| | CTCF HiChIP GM12878 | 5419 | 25219 | 11494 | 11529 | 978 | 1897 | 2029 | 1001 | 178 |
| | SMC1 HiChIP HCC1599 | 698 | 5800 | 1905 | 1962 | 54 | 142 | 147 | 46 | 16 |
| | SMC1 HiChIP REC-1 | 814 | 6034 | 6034 | 2184 | 92 | 205 | 241 | 93 | 25 |
| | | | | | | | | | | |
| ChIA-PIPE | SMC1 dcHiChIP HG00731 | 9354 | 30128 | 16943 | 16825 | 14237 | 23015 | 23489 | 14059 | 31456 |
| | CTCF HiChIP HG00731 | 4110 | 11975 | 6886 | 6669 | 2730 | 4498 | 4611 | 2554 | 2074 |
| | SMC1 HiChIP GM12878 | 3941 | 7175 | 5228 | 5152 | 9472 | 11899 | 12141 | 9304 | 27926 |
| | CTCF HiChIP GM12878 | 20282 | 45461 | 30781 | 30124 | 18009 | 45461 | 28691 | 18046 | 16899 |
| | SMC1 HiChIP HCC1599 | 1637 | 2574 | 1876 | 1871 | 1848 | 2103 | 2209 | 1892 | 3037 |
| | SMC1 HiChIP REC-1 | 3392 | 5160 | 4037 | 4005 | 4364 | 5307 | 5475 | 4643 | 9719 |

**Supplementary Table 6**. CTCF motif orientation analysis at HiChIP contact anchors in all the samples detected by 3 different loop calling algorithm - nf-HiChIP, HiCCUPS and ChIA-PIPE.

| Loop calling pipeline | Dataset | EP Loops | PP Loops | % EP+PP Loops |
|---|---|---|---|---|
| nf-HiChIP | SMC1 dcHiChIP HG00731 | 27495 | 8303 | 44.4767478 |
| | CTCF HiChIP HG00731 | 10392 | 3295 | 48.86120234 |
| | SMC1 HiChIP GM12878 | 13025 | 3428 | 45.03352949 |
| | CTCF HiChIP GM12878 | 60680 | 16942 | 43.47718935 |
| | SMC1 HiChIP HCC1599 | 5449 | 1089 | 38.1803317 |
| | SMC1 HiChIP REC-1 | 7679 | 2031 | 43.55431955 |
| | | | | |
| HiCCUPS | SMC1 dcHiChIP HG00731 | 13912 | 4630 | 42.97302308 |
| | CTCF HiChIP HG00731 | 9659 | 3657 | 50.07897706 |
| | SMC1 HiChIP GM12878 | 6579 | 2416 | 46.18030599 |
| | CTCF HiChIP GM12878 | 17845 | 5196 | 38.56040701 |
| | SMC1 HiChIP HCC1599 | 3571 | 1342 | 45.57936729 |
| | SMC1 HiChIP REC-1 | 4107 | 1539 | 47.96126402 |
| | | | | |
| ChIA-PIPE | SMC1 dcHiChIP HG00731 | 45198 | 12131 | 31.93531496 |
| | CTCF HiChIP HG00731 | 12585 | 2940 | 33.66437539 |
| | SMC1 HiChIP GM12878 | 20401 | 4033 | 26.48729512 |
| | CTCF HiChIP GM12878 | 57597 | 10827 | 28.87125153 |
| | SMC1 HiChIP HCC1599 | 3601 | 1379 | 26.13212993 |
| | SMC1 HiChIP REC-1 | 8663 | 1199 | 21.3870576 |

**Supplementary Table 7**. Number of Enhancer-Promoter (EP) and Promoter-Promoter (PP) loops and percentge of combined EP and PP loops identified for all HiChIP samples from 3 different loops calling algorithm - nfHiChIP, HiCCUPS and ChIA-PIPE.

| **Dataset** | **Stripe calling algorithm** | | | |
|---|---|---|---|---|
| | Stripenn | gStripe, using loop set from: | | |
| | | nf-HiChIP | HiCCUPS | ChIA-PIPE |
| SMC1 dcHiChIP HG00731 | 3603 | 4187 | 6237 | 18155 |
| SMC1 HiChIP GM12878 | 3908 | 1526 | 1479 | 9606 |
| SMC1 HiChIP HCC1599 | 1136 | 757 | 497 | 2100 |
| SMC1 HiChIP REC-1 | 1637 | 1223 | 424 | 6458 |

**Supplementary Table 8.** Number of stripes called in each dataset.

| Parameter | | Dataset | | | |
|---|---|---|---|---|---|
| canny | bfilter | SMC1 dcHiChIP HG00731 | SMC1 HiChIP GM12878 | SMC1 HiChIP HCC1599 | SMC1 HiChIP REC-1 |
| 1 | 1 | 3390 | 3023 | 567 | 563 |
| | 3 | **3603** | 3703 | 840 | 1201 |
| | 5 | 3072 | **3908** | 1088 | 1599 |
| 1.5 | 1 | 3440 | 3871 | 968 | 1470 |
| | 3 | 2797 | 3753 | 1042 | **1637** |
| | 5 | 1904 | 3534 | 1080 | 1541 |
| 2 | 1 | 2102 | 3413 | **1136** | 1597 |
| | 3 | 1778 | 3488 | 1090 | 1565 |
| | 5 | 1111 | 2933 | 944 | 1349 |
| 2.5 | 1 | 1084 | 2779 | 972 | 1321 |
| | 3 | 947 | 2522 | 885 | 1220 |
| | 5 | 603 | 2230 | 744 | 882 |
| 3.0 | 1 | 466 | 2051 | 731 | 879 |
| | 3 | 414 | 1903 | 655 | 826 |
| | 5 | 236 | 1590 | 540 | 620 |

**Supplementary Table 9.** Number of stripes called by Stripenn in each dataset, using the particular combinations of *canny* (Canny edge detection parameter) and *bfilter* (kernel size of the mean filter) parameters (see: Yoon et al. 2022). The default values are: 2.0 for canny and 3 for bfilter. The maximum values in each dataset is marked in bold.
