# Supplementary material for: Improved cohesin HiChIP protocol and bioinformatic analysis for robust detection of chromatin loops and stripes
Source: Commun Biol. 2025 Mar 14;8:437. doi: 10.1038/s42003-025-07847-w (PMC11906747; doi:10.1038/s42003-025-07847-w)
Supplement: Supplementary file 3 — Reporting Summary [file 42003_2025_7847_MOESM3_ESM.pdf]

nature portfolio

Corresponding author(s): Dariusz Plewczynski, Karolina Buka

Last updated by author(s): Feb 19, 2025

Reporting Summary

Nature Portfolio wishes to improve the reproducibility of the work that we publish. This form provides structure for consistency and transparency in reporting. For further information on Nature Portfolio policies, see our Editorial Policies and the Editorial Policy Checklist.

Statistics

For all statistical analyses, confirm that the following items are present in the figure legend, table legend, main text, or Methods section.

n/a	Confirmed	
☒	☐	The exact sample size (n) for each experimental group/condition, given as a discrete number and unit of measurement
☒	☐	A statement on whether measurements were taken from distinct samples or whether the same sample was measured repeatedly
☒	☐	The statistical test(s) used AND whether they are one- or two-sided<br>*Only common tests should be described solely by name; describe more complex techniques in the Methods section.*
☒	☐	A description of all covariates tested
☒	☐	A description of any assumptions or corrections, such as tests of normality and adjustment for multiple comparisons
☒	☐	A full description of the statistical parameters including central tendency (e.g. means) or other basic estimates (e.g. regression coefficient) AND variation (e.g. standard deviation) or associated estimates of uncertainty (e.g. confidence intervals)
☒	☐	For null hypothesis testing, the test statistic (e.g. F, t, r) with confidence intervals, effect sizes, degrees of freedom and P value noted<br>*Give P values as exact values whenever suitable.*
☒	☐	For Bayesian analysis, information on the choice of priors and Markov chain Monte Carlo settings
☒	☐	For hierarchical and complex designs, identification of the appropriate level for tests and full reporting of outcomes
☐	☒	Estimates of effect sizes (e.g. Cohen's d, Pearson's r), indicating how they were calculated

Our web collection on statistics for biologists contains articles on many of the points above.

Software and code

Policy information about availability of computer code

Data collection	No software used for data collection
Data analysis	Zendo -<br>Link - https://zenodo.org/records/11213539<br>DOI - 10.5281/zenodo.11213538<br><br>Github -<br>nf-HiChIP pipeline -https://github.com/SFGLab/nf-hichip<br>gStripe algorithm -https://github.com/SFGLab/gStripe<br>LoopSage algorithm -https://github.com/SFGLab/LoopSage<br><br>Requirments -<br>#nf-HiChIP -<br>scipy>=0.17.0<br>py2bit>=0.1.0<br>pyBigWig>0.2.1<br>matplotlib>=1.4.0<br>pandas==v0.20.3<br>numpy==v1.17.1<br>pysam==v0.15.2<br>pybedtools==v0.8.0

Cython
click
#Post-Processing
Pickle ==v3.12.4
pandas ==2.2.2
numpy==2.0.0
matplotlib==3.9.0
matplotlib-venn== 0.11.10
#gSTRIPE
numpy >= 2.0.0
pandas >= 2.2.2
scipy >= 1.13.1
igraph >= 0.11.5
matplotlib >= 3.9.0
setuptools>=70.1.0
seaborn >= 0.13.2
#LoopSage
scipy>=1.11.4
mdtraj>=1.9.9
seaborn>=0.13.0
statsmodels>=0.14.0
matplotlib>=3.8.2
numpy>=1.26.2
pandas>=2.1.3
pybigwig>=0.3.22
imageio

Other software used for data analysis-
Juicer (https://github.com/aidenlab/juicer)
ChIA-PIPE (https://github.com/TheJacksonLaboratory/ChIA-PIPE)
MACS (https://github.com/macs3-project/MACS)
deepTools (https://github.com/deeptools/deepTools)

Software Used for data visualization -
IGV (https://igv.org/)
Juicebox (https://github.com/aidenlab/Juicebox)

For manuscripts utilizing custom algorithms or software that are central to the research but not yet described in published literature, software must be made available to editors and reviewers. We strongly encourage code deposition in a community repository (e.g. GitHub). See the Nature Portfolio guidelines for submitting code & software for further information.

Data

Policy information about availability of data

All manuscripts must include a data availability statement. This statement should provide the following information, where applicable:

- Accession codes, unique identifiers, or web links for publicly available datasets
- A description of any restrictions on data availability
- For clinical datasets or third party data, please ensure that the statement adheres to our policy

Sequencing data generated in this study is deposited in the Gene Expression Omnibus (GEO) database, with an accession number GSE266640.

Research involving human participants, their data, or biological material

Policy information about studies with human participants or human data. See also policy information about sex, gender (identity/presentation), and sexual orientation and race, ethnicity and racism.

Reporting on sex and gender	n/a
Reporting on race, ethnicity, or other socially relevant groupings	n/a
Population characteristics	n/a
Recruitment	n/a
Ethics oversight	n/a

Note that full information on the approval of the study protocol must also be provided in the manuscript.

Field-specific reporting

Please select the one below that is the best fit for your research. If you are not sure, read the appropriate sections before making your selection.

☒ Life sciences ☐ Behavioural & social sciences ☐ Ecological, evolutionary & environmental sciences

For a reference copy of the document with all sections, see nature.com/documents/nr-reporting-summary-flat.pdf

Life sciences study design

All studies must disclose on these points even when the disclosure is negative.

Sample size	For each HiChIP experiment 10 millions cells were used
Data exclusions	No data was excluded
Replication	HiChIP experiments were performed in two biological replicates
Randomization	Since we did not use different experimental groups, randamization was not necessary
Blinding	Blinding was nor revelant because of our experinmental design

Reporting for specific materials, systems and methods

We require information from authors about some types of materials, experimental systems and methods used in many studies. Here, indicate whether each material, system or method listed is relevant to your study. If you are not sure if a list item applies to your research, read the appropriate section before selecting a response.

Materials & experimental systems

n/a	Involved in the study	
☐	☒	Antibodies
☐	☒	Eukaryotic cell lines
☒	☐	Palaeontology and archaeology
☒	☐	Animals and other organisms
☒	☐	Clinical data
☒	☐	Dual use research of concern
☒	☐	Plants

Methods

n/a	Involved in the study	
☒	☐	ChIP-seq
☒	☐	Flow cytometry
☒	☐	MRI-based neuroimaging

Antibodies

Antibodies used	SMC1 antibody (Bethyl Laboratories, A300-055A)<br>CTCF antibody (Abclonal, A1133)
Validation	Both antibodies were validated in human lymphoblastoid cell line (LCL) by ChIP-qPCR experiments. They were also validated by WB by the manufacturers as indicated on their websites and they have been used in previous studies for HiChIP experiments (Mumbach et al., 2016; Mumbach et al., 2019)

Eukaryotic cell lines

Policy information about cell lines and Sex and Gender in Research

Cell line source(s)	HG00731 LCL was purchased from the Coriell Institute
Authentication	Cells were purchased directly from the Coriell Institute and were not further authenticated.
Mycoplasma contamination	Cell were tested negative for mycoplasma contamination
Commonly misidentified lines<br>(See ICLAC register)	None

Plants

Seed stocks	NA
Novel plant genotypes	NA
Authentication	NA
